# Exploiting the “video game craze”: A case study of the tobacco industry’s use of video games as a marketing tool

**DOI:** 10.1371/journal.pone.0220407

**Published:** 2019-07-25

**Authors:** Patricia A. McDaniel, Susan R. Forsyth

**Affiliations:** Department of Social and Behavioral Sciences, School of Nursing, University of California, San Francisco, San Francisco, California, United States of America; University of Milan, ITALY

## Abstract

**Background:**

Video games have grown in popularity since the 1970s, and tobacco imagery is present in a substantial subset of games, including those oriented to youth. Much like exposure to tobacco content in films, exposure to tobacco content in video games may influence smoking uptake and use; however, the tobacco industry’s role in facilitating or promoting the use of tobacco imagery in video games is unclear. We explored the industry’s interest in and use of video games to market their products to youth and young adults.

**Methods:**

We retrieved and analyzed archival tobacco industry documents. We supplemented information from the documents with current and archived versions of several brand and corporate websites and one website containing user-supplied information on video games.

**Results:**

Tobacco companies recognized the youth appeal and marketing potential of video games as early as 1980. Initial marketing ideas included incorporating video game themes into product packaging and design. More fully realized plans focused on incorporating video games into product promotions in bars, as a high visibility way to attract younger patrons and increase long-term marketing opportunities by generating names for tobacco company direct-marketing databases. Tobacco companies also incorporated video games into in-home product promotions, primarily as components of brand websites, in order to enhance brand image and generate repeat website traffic. A similar desire to attract and keep visitors led to discussions about the inclusion of video games on corporate youth smoking prevention websites, although only one company, Lorillard, followed through.

**Conclusions:**

Video game players are an attractive target market for tobacco companies. Video games, as used by these companies, facilitate consumer engagement with particular tobacco brands or particular corporate messages. Eliminating the use of video games as a promotional vehicle may require limiting tobacco marketing in both physical and online environments.

## Introduction

Since the 1970s, video games have grown in popularity, such that the video game industry now generates three times the revenue of the film industry [1]. Although regarded as a youthful pastime, in the US, video game play is also common among adults (ages 30–49), with boys and young men predominating in both the younger and older player age groups [2, 3]. Since it first appeared in 1978 [4], advertising in video games has become common [5], likely due video games’ popularity among youth and adults, key target markets [6], and to advertising’s potential impact: in-game exposure to brands is memorable [7] and creates positive brand associations [8]. Many consumer product companies have embraced the concept, including Budweiser [9], Coca-Cola, and Subway [10].

The tobacco industry has long been a marketing innovator, inventing marketing media [11] and overcoming marketing restrictions [12]. Tobacco imagery is present in a substantial subset of video games played by adolescents, including those rated as “teen appropriate” by the Entertainment Software Ratings Board [13–17]. This imagery includes branded and unbranded tobacco products (e.g., discarded cigarettes, advertisements for fictional cigarette brands) and characters with lit cigarettes or cigars in their mouths or cigarette packs rolled into shirtsleeves [14–16]. The impact on adolescent smoking initiation of exposure to this imagery is unclear due to limited research and inconclusive results [14]. Some studies, for example, have found an association between video game playing and smoking, while others have not [14, 18]; however, most suffer from methodological flaws, such as grouping video game playing with television or internet use, and small or non-representative samples [14].

There are several reasons to expect an association between exposure to tobacco imagery in video games and smoking initiation. Exposure to smoking scenes in movies is causally related to youth smoking behaviors, with the relationship explained through theories of media influence [15]: smoking in movies influences adolescent beliefs about the social acceptability and popularity of smoking, beliefs strongly linked to smoking initiation [15, 19, 20]. Viewing tobacco imagery in video games may have a similar impact on adolescent beliefs about smoking, and ultimately, on smoking behavior. Video games may also have a more direct impact on youth smoking. Engaging in virtual behaviors has been found to modify real-world behavior, with video games offering adolescents opportunities to try behaviors, including smoking, or identities, such as “smoker,” that are rewarded or glorified in the game, and which then transfer to the real world [21–23].

Tobacco companies have been known to create their own video games [24] and to include video games on tobacco brand websites [25, 26]. Yet researchers have not fully explored the tobacco industry’s use of video games as a marketing tool. This paper uses internal tobacco industry documents to examine how and why tobacco companies have employed video games to market their products to youth and adults from the 1980s onwards.

## Methods

As a result of litigation, nearly 15 million previously undisclosed industry documents [27, 28] are archived in a full-text searchable electronic repository [29]. We searched the archive using standard tobacco industry document search strategies [28, 30, 31], which include starting with broad search terms (“video games,” “online gaming”) and using retrieved documents to identify more specific terms. Our initial broad search terms led to search terms related to brand-related video games (e.g., Newport video game; Doral bingo); games developed for bar nights (e.g., Ultimate Powder; Sega racing simulators); games developed for brand websites (e.g., Camel casino; smokerswelcome.com); youth smoking prevention (e.g., buttoutnow.com; Crisis Crew); and game designers (e.g., Lazertron; Media Circus). These search terms returned thousands of documents. To narrow our findings, we excluded duplicates and documents unrelated to our topic of interest (such as tobacco company marketing activities that did not incorporate a video game component). We located additional relevant documents using a search feature that allows for the identification of documents with adjacent reference (Bates) numbers (sequential numbers stamped on every page as documents are produced in litigation; documents with sequential numbers are frequently related). We stopped searching when additional searches yielded no new documents.

We identified 293 documents that were relevant to our focus on tobacco companies’ interest in video games as a marketing tool; their dates ranged from 1980 to 2008. These included marketing and promotion documents, contracts between tobacco companies and video game makers, tobacco company requests for proposals for video game designs, internal research on consumer behavior related to playing video games, and youth smoking prevention materials. For additional contextual information, we examined current and archived versions of several brand and corporate websites (e.g., Camel.com, buttoutnow.com), and the website Mobygames.com, an online database with user-supplied information on video games, including, for example, promotional and user screenshots.

We analyzed documents using a hermeneutic approach. Broadly, the term “hermeneutic” means interpretation of text, in which one tries to appraise the meaning of the material within the context of the time and place in which it was produced [32–35]. This approach involves identifying themes and thematic clusters, triangulating documentary data by looking for consistency among sources, and employing reliability and validity checks, by, for example, seeking investigator agreement regarding the interpretation of documents [36].

Consistent with this hermeneutic approach, we did not seek to test specific hypotheses about the tobacco industry’s use of video games as a marketing tool [37–39]. Instead, to develop this interpretive account, the first author reviewed all documents and website material and took detailed notes, and both authors reviewed selected key documents. We relied upon iterative reviews of the documents and our notes to identify and evaluate common themes, construct a timeline of events, and assemble a case study [32, 40]. We cite 97 tobacco industry documents in the text; a supplemental file contains a list of all relevant documents.

## Results

### Initial ideas for capitalizing on the “video game craze”

In the 1980s, tobacco companies explored ways to capitalize on the “widespread video game craze” [41]. For example, in 1980, RJ Reynolds (RJR) tested the feasibility of a video game tournament as a promotional vehicle for its Camel brand among young adults [42]. (“Young adult” is the term that the industry began using in the 1970s in place of the more politically sensitive “youth,” irrespective of any social psychological definition of young adulthood) [43]. An RJR employee noted that video games are among the fasting growing entertainment media in the world. Their popularity is particularly strong within the young adult segment of the marketplace. Video games have not yet been tapped by consumer package good companies seeking young adult loyalty. This puts the CAMEL family and [RJR] in a pre-emptive position [42].

RJR apparently did not pursue this idea to be the first tobacco company to use video games to market cigarettes to young adults, or at least not overtly: two years, later, in an October 1982 “idea session,” Lorillard personnel noted that “The widespread video game craze has certain fundamental features which *we could be the first to exploit*” (emphasis added) [41]. Their suggestions were to incorporate video game imagery into pack design, or use product names such as “Galaxy,” “Cosmos,” and “Universe” to capitalize on space-themed video game popularity [41]. While these ideas were apparently rejected, in 1983, Lorillard took a page from RJR’s book, adding video game contests (using existing games) to its spring break Newport promotion in Daytona Beach, Florida [44].

In 1985, RJR returned to the video game theme as it tried to design brands to compete with Marlboro among youth [45, 46]. Marketing ideas for the new brands included video games as promotional vehicles, as branded games [47] or as “an advertising medium in bars/clubs” [48]. RJR also considered marketing existing brands near “video game arcades,” where “younger-adult smokers ‘hang out’” [49].

### Tobacco company bar promotions incorporate video games

In the 1980s, tobacco companies began incorporating video games into product promotions in bars. Because bars restricted entry to those age 21 and over, they offered tobacco companies the opportunity to promote their products to young adults while avoiding criticism that they were targeting children [50]. The goals of bar promotions were to create and reinforce brand image; market to young adults in a smoker-friendly environment; encourage product trial; increase brand visibility; and generate names and addresses for companies’ direct mail databases [50].

Because bar promotions already included party games (e.g., trivia contests, puzzles, and card games) [51–54], adding video games to the mix fit with bar night activities. However, video games offered advantages over party games. For example, they might include a “bright” “lit up” screen, which, in the words of a Philip Morris (PM) marketing manager, if seen from across the room, “will draw [the] attention [of] adult smokers to come over and participate” [55]. According to PM, due to their popularity and “high tech” nature, video games also generated more excitement, interest, and traffic [56, 57] than traditional party games [58] among young adults, who PM considered “extremely technology savvy” and eager to use new technology [58].

PM incorporated video games into its bar promotions (chiefly for its Marlboro brand) from the early 1990s onwards. For example, its Formula 1 racing-themed bar promotions (tied to its sponsorship of a Formula 1 team) included virtual racing simulators and laptop-based video games. Video game maker Sega manufactured PM’s first 4 simulators [59], in which players raced to finish a computer-generated course, complete with physical cues: “Hit a retaining wall and your cockpit shudders. Take a turn too fast and an airbag inflates at your side, simulating the G-forces that push real drivers around in the saddle. Brake hard and the seat pitches you forward” [60]. To enable “easy viewing by spectators,” three 19” monitors were positioned above the simulators [61]. There was Marlboro branding on the cars and the monitor frames “to create added excitement and on-site awareness” [60, 62]. Winners received branded merchandise (t-shirts, watches, etc.) [63], until 1999, when the Master Settlement Agreement prohibited such activity. To participate, bar patrons were required to fill out a “survey” that included their name, address, date of birth, gender, current and previous brand preferences, purchase patterns, and contact information for other smokers “willing to receive free cigarettes and incentive items in the mail” [63]. A journalist observing players noted that the simulator was compelling enough that nonsmokers also “filled out the survey to say that they [smoked] in order to play the video game” [64].

After determining that the simulators were a stronger draw than laptop-based video games [59], PM spent $712,652 between 1997 and 2001 for 24 simulators to expand racing-themed bar promotions. These simulators used an existing computer-based game, IndyCar Racing II, made by Sierra Papyrus, with the software customized to include Marlboro logos on the cars [65] and a “branded cowling to cover the [computer] monitor and pedestal” [66]. In 1997, 104,400 people attended bar nights with racing simulators (approximately 300 at each event), generating 13,100 names for PM’s direct marketing database (78% of which were new) [67].

PM also demonstrated interest in developing a virtual reality game for Marlboro promotions, one that would “immerse players in a 3-D generated computer world” [68]. As described in a consultants’ presentation, a virtual reality game offers a tremendous ‘crowd attractant’ factor. Since the technology is unique and not always accessible, it attracts people who have not previously been able to experience it. … [It] commands very high visibility and garners extraordinary FREE media/publicity exposure–everyone will want to see the hottest thing traveling through town. … There is no doubt about the positive brand association which will impact not only participating consumers, but [also] people they tell about their exciting experiences [68].

In September 1995, PM agreed to pay game designers Media Circus approximately $500,000 to create a 4 minute, 3-dimensionsal, virtual reality, Marlboro branded driving experience” [69, 70] for bar nights [71–74]. PM had high hopes for the game: the kickoff meeting summary stated that “We’re counting on this game to be the hit of the night …[It] needs to be huge to represent the mega brand image” [75]. It was expected to have a “Disney like impact … a very big … experience in a small amount of space” [75]. This document also noted that they had replaced the descriptor “game” with “promotion,” since “PMUSA does not promote to kids and [there is] sensitivity to ‘game’ internally…don’t want to trivialize the … promotion” [75]. However, PM canceled the project several months later [76], possibly because of cost considerations [77].

Brown and Williamson (BW), whose KOOL brand sponsored auto racing in the late 1990s and early 2000s, invested approximately $450,000 in racing simulators for its KOOL bar nights [78]. Eight teams traveled the US visiting 42 cities for 4–5 week stays, holding bar nights 3–4 times per week, with two racing simulators providing an “‘in your face’ street racing experience” [79–81]. The program’s goal was to “enhance contemporary brand image for KOOL” [82] particularly among young men [83]. The simulators were intended to help KOOL “own the evening” [84]. Young men who were surveyed about their KOOL bar night participation reported that the racing simulator event was “more fun than other sponsored bar nights”; 36% reported that their impression of KOOL was more favorable after the event [82].

Video games at tobacco company-sponsored bar nights were not focused exclusively on auto racing. For example, in 2000, PM paid Lazertron (later, Arcade Planet) $266,444 for 64 video game consoles and 64 copies of a specially-created Marlboro-branded downhill skiing game, “Ultimate Powder” [85]. The game’s objective was to avoid obstacles and ski to the Marlboro party as quickly as possible [86]; it was used at bar nights promoting “Winter Ranch” sweepstakes, whose winners spent time at a Marlboro-themed Montana ranch [87]. In 2001, PM sought proposals to create 200 touch screen interactive kiosks that would host multiple video games and enable multi-player play [88]. The goal was to “provide a high visibility branding unit in a bar” that would “generate interest” among “tech savvy” young adult smokers (YAS) and “create talk value” about Marlboro events [58, 88, 89]. Design specifications noted that “screens should draw attention of YAS–use bright colors etc.” [58]. Potential video games were western/ranch themed (e.g., “cowboy checkers,” “wagon train”), in keeping with broader Marlboro advertising, and contained Marlboro branding on the introductory and exit screens [58, 90, 91]. (It is unclear whether these kiosks were ever created).

RJR planned to launch a new Winston bar program in 2001, an update on the brand’s 1996 “No Bull. No Boundaries” repositioning [92]. It featured virtual reality units that allowed players to experience sky diving, shark feeding, piloting a fighter jet, etc., in a fully branded Winston environment [92]; real-life versions of these experiences were offered to Winston sweepstakes winners [92]. RJR concluded that its target market, 21–34 year olds who “enjoy extreme activities and the thrill of life” [92] was a “perfect” match for virtual reality technology: “The mere sight of someone wearing a headset generates tremendous levels of interest” [93]. It sought bids from two companies, cautioning that games should be “durable” and simple enough for “intoxicated patrons” [93]. It is unclear whether any games were created; a 2002 proposal to consider a virtual reality concept “like nothing you have ever seen” for the 2003 Winston bar program suggested that RJR had not yet committed to the technology [94]. The cost may have been prohibitive: one bid was nearly $550,000 for one simulator (with software) [95].

### Video games as in-home promotional tools

Tobacco companies also incorporated or considered incorporating video games into in-home product promotions. In 1999, as personal computer ownership was becoming more common, RJR offered Doral smokers steep discounts on video games on CD-ROMs (e.g., Pro Bass Fishing, Beat the House II) with proofs-of-purchase [96, 97]. The goal was to “attract younger adult smokers (ages 21–35),” changing their image of the brand “while rewarding existing customers” [98]. The promotion was deemed a success, with more CD-ROMs requested than anticipated [99]. Marketing consultants recommended that RJR consider a similar promotion for Winston, partnering with a game developer to create a NASCAR-themed game (with minimal or expansive Winston branding) [99]. The consultants noted that the “opportunity exists for Winston to pre-empt P[hilip] M[orris] on the use of software in promotions” [99]. Initially, two developers “declined to participate with a tobacco company,” but after “discussions with RJR and NASCAR top management,” one reconsidered [99]. The company, Papyrus, released a NASCAR-themed game in 2001 that referenced the Winston cup (including in a billboard inside the game) but it is unclear whether it included other Winston advertising [100].

In 2004, BW’s KOOL Mixx promotion included a CD-ROM with games “to test your hip hop skills,” distributed in magazines such as *Rolling Stone* and *Vibe* [101]. The company was sued by various attorneys general for targeting youth in violation of the Master Settlement Agreement [101], and initially agreed to stop distributing the CD-ROM [102]; RJR, which acquired BW, agreed to a more comprehensive settlement, including limiting distribution of any future CD-ROMs to "adult-only" facilities and by mail to known adult smokers [103].

In the early 2000s, tobacco companies began creating brand websites, which represented a “powerful new marketing vehicle” that could “bring brand positioning to life” through “sight, sound, [and] motion” [104]. Among the first was RJR’s Doral website, which included four games, bingo, skeet shooting, crosswords, and bowling [105]. Other brand websites also considered including video games as a way to “generate repeat traffic” [106]. By 2005, all of RJR’s major brands (Camel, KOOL, Winston, Salem, etc.) had websites [107] and several featured games tied to brand promotions. For example, the 2005 Camel website featured “Camel Casino,” which allowed players to play games such as blackjack and poker [108]; poker “chips” were distributed on packs, at events, via direct marketing, and via email [109], with the goal of “driv[ing] adult smokers to the brand’s website” for prize redemption or competition in multi-player or individual games [110]. The company that partnered with RJR on the games, Arkadium, noted that “interactive online games engage consumers with brands in a way never before possible,” with players “devot[ing] significant time and emotional investment in games” [111]. From 2007–2008, Camel Casino was the second most popular section of Camel’s website, after the home page, drawing 591,542 visits [112].

KOOL had a “Play on the House/Spades Slam” promotion at retail and in bars in 2002; in 2003, it added an online component that allowed website visitors to play spades against the computer or other online players [113]. Consultants hired by BW to examine the website’s effectiveness interviewed 125 smokers aged 21–29 who had registered for and spent at least 3 minutes on the website [113]. 33% spent more than 30 minutes on the website; 23% visited 6 or more times [113]. The game was the most popular website feature, the “anchor” for website activity, [and had a positive impact on KOOL brand image and purchase among those who usually smoked other brands [113].

As tobacco companies began creating youth smoking prevention (YSP) programs in the late 1990s [114], several considered building YSP-branded websites for kids, complete with video games, a “traffic driver” for kids’ sites [115]. PM, for example, hired consultants in 1998 and 1999 to propose website options for its Think…Don’t Smoke YSP program [116, 117]. Possibilities included creating a new website with a “Nascar style racing game with ‘Don’t Smoke’ messages built in (e.g., in leader board)” [118]. One PM consultant raised the issue of capturing kids’ personal information, and recommended requiring parental permission to use any PM-sponsored YSP website [119]. The consultant also pointed out that a decision to create a PM-sponsored website would mean that PM was taking responsibility for “privacy, security, data capture, parental permission, monitoring” and for “marketing expertise about kids (in fact, this will be implicit just by having such a site)” [119]. PM continued exploring the idea of a YSP program web presence, complete with sports-themed video games (snowboarding, BMX bike riding) [120]; however, it ultimately chose not to create one.

Lorillard, by contrast, moved forward with a website for teens in December 1999, buttoutnow.com, to complement its “Tobacco is Whacko if You’re a Teen” YSP program. The goal of the YSP program was to “address the youth smoking issue in a responsible and *visible* manner” [121] (emphasis added), and a web presence, with games, helped advance that goal by “keep[ing] kids at the site” [122]. The website initially included two video games, “the Search,” and “Crisis Crew” [123]. The Search involved searching for missing puzzle pieces in a poster featuring the Tobacco is Whacko character [124, 125]. The object of Crisis Crew was to avoid a smoking dragon (and demonstrate the value of teamwork and leadership) by assigning various tasks to a group of kids (e.g., building ladders or sailing a ship) [125–127].

The available documents suggest that parental permission was not required for website access, although the privacy policy recommended that visitors aged 13 to 17 “seek parental consent before providing any [personally identifiable information]” [128]. After one year, the website had drawn nearly 600,000 visitors [129], with the games ranking second in popularity, well behind the top attraction, information on Lorillard’s college scholarship program [130]. Both games were replaced in 2002 to “increase traffic and repeat visitors” [131]. The replacement game featured a skateboarder who fought “against time, physics, and obstacles to spread the anti-smoking message” [132–134]. Traffic did increase in 2002, with the college scholarship pages still the most popular portion of the website [132]. The website appears to have been active until April 2004 [135]. Although exposure to television advertisements for Lorillard and PM’s YSP programs was found to be associated with stronger youth approval of smoking and intentions to smoke, weaker perceptions of smoking-related harms, and greater likelihood of current smoking [136], research has not explored whether the inclusion of video games on Lorillard’s website had a similar impact on youth.

## Discussion

Tobacco companies recognized the marketing potential of video games early in the history of the “video game craze.” Although initial ideas for how to capitalize on this craze included creating packaging and brand names evocative of video games and advertising existing brands near video game arcades, tobacco companies settled on more immersive and interactive marketing strategies. These included adding video game components to bar nights, a common strategy across companies, which saw video games as a way to attract “tech savvy” young adults’ attention and interest, thereby persuading smokers (and in some cases nonsmokers) to provide personal information for tobacco company marketing databases in order to play the games. Inclusion in these databases put participating bargoers at risk: the receipt of direct mail coupons from tobacco companies is associated with smoking initiation among nonsmokers and continued smoking among smokers [137, 138].

Video games remain a feature of tobacco company bar nights and traveling “lounges” [139, 140]. It is unclear how large of a draw they are, given that consumers now have greater access to video games than they did in the 1990s; however, it seems unlikely that video games would remain a component of these events if they no longer advanced tobacco company goals. Recent technological advances and reduced costs associated with virtual reality games may enable tobacco companies to replace standard video games with a type of game to which they attached high hopes in the mid-1990s and early 2000s. If it met expectations, the fully-branded, immersive, “Disney-like” experience of virtual reality would be a big draw—particularly given the expense of purchasing the technology oneself and the limited content available [141]. “Disney-like” popularity would ensure the continued growth of tobacco company direct marketing databases, facilitating long-term exposure to tobacco marketing.

Tobacco companies incorporated video games into in-home brand marketing and promotion efforts as well. Video games (both branded and unbranded) were an appealing component of these promotions because they promised to attract a younger audience—an appeal recognized by the attorneys general who brought legal action against BW for its “KOOL Mix” promotion. Branded video games also offered the potential for recurring brand exposures to players of all ages through repeat playing, a feature absent from video games played at bar nights, and one with direct implications for smoking behavior. Although research exploring the relationship between viewing tobacco imagery in video games and subsequent tobacco-related behavior is still in its infancy [14], there is an established causal relationship between exposure to tobacco marketing in many different media and youth smoking initiation and progression to regular smoking [15]. Exposure to tobacco marketing for even a limited time can create positive perceptions of smoking and smokers and influence intentions to smoke among youth [15]. Thus, repeat exposure to tobacco imagery in video games played at home is a critical cause for concern.

Tobacco companies also relied on the popularity of video games to draw visitors to brand and corporate websites. As others have noted, websites offer tobacco companies distinct advantages over traditional media in terms of interactivity, length of exposure, and avoidance of marketing restrictions [25, 142]. Video games offer similar advantages, and are thus a natural fit for tobacco brand websites. On brand websites, video games were not only among the most popular features, but, as suggested by internal research, their inclusion may help achieve two perennial tobacco company marketing goals: improving brand image and influencing purchasing decisions. Online video games–which continue to be a feature of tobacco brand websites [25, 26]—thus represent a powerful, and, for the moment, largely unregulated tobacco marketing tool. The same is true of online games on tobacco companies’ corporate websites. This appears to be less of a concern at the moment, however. Lorillard was the only tobacco company willing to proceed with a website designed specifically for youth, apparently unconcerned about any potential controversy over collecting data about children that could be used for marketing purposes. This website became inactive in 2004, and we are unaware of other tobacco companies sponsoring similar websites for youth.

In the US, the Master Settlement Agreement places limits on paid tobacco product placement in video games. Our study found no evidence that tobacco companies paid for product placement; instead, the documents we uncovered point to more direct relationships, with tobacco companies commissioning branded games that promoted a particular brand (or corporate) image. Addressing these relationships and eliminating the use of video games as a promotional vehicle may require an approach that focuses on limiting tobacco marketing in both physical and online environments. Indeed, the WHO Framework Convention on Tobacco Control recommends prohibiting “any form of commercial communication … with the aim, effect or likely effect of promoting a tobacco product or tobacco use either directly or indirectly” [143]. In the US, the 2009 Family Smoking Prevention and Tobacco Control Act gives the Food and Drug Administration broad authority to regulate tobacco marketing in order to promote overall public health [144]. Thus, in some jurisdictions, it may be possible to restrict tobacco branding or other tobacco imagery in video games. Media campaigns to denormalize tobacco content in video games and more focused efforts to capitalize on some game makers’ apparent reluctance to work with tobacco companies, as occurred with RJR in the 1990s, could also be considered.

Our study provides only a partial understanding of the tobacco industry’s use of video games as a marketing tool. Due to the size of the document databases, we may not have retrieved every relevant document. Some may have been destroyed or concealed by tobacco companies, including, for example, any documents concerning payments for tobacco product placement in video games; others may have never been obtained in the legal discovery process.

## Conclusions

Since the activities outlined here, video games have become more sophisticated, ubiquitous, and global, and tobacco content in games has become commonplace [145]. Video game players remain an attractive target market for tobacco companies, and we can expect continued tobacco industry interest in using games to attract and engage consumers. Given the established causal link between exposure to tobacco content in films and youth smoking initiation [15, 146], the tobacco industry’s continued interest in video games as a marketing tool poses a challenge to tobacco control. Strategies to address this challenge are needed.

## Supporting information

S1 Bibliography List of all relevant documents retrieved. (DOCX)
