# Supplementary material for: Exploiting the “video game craze”: A case study of the tobacco industry’s use of video games as a marketing tool
Source: PLoS One. 2019 Jul 25;14(7):e0220407. doi: 10.1371/journal.pone.0220407 (PMC6657901; doi:10.1371/journal.pone.0220407)
Supplement: S1 Bibliography — (DOCX) [file pone.0220407.s001.docx]

S1. Bibliography. List of relevant documents retrieved

1. Howard D. Marlboro Bar Night Interactive Kiosk Interactive Kiosk Creative Brief 2002. February 02 2001. Philip Morris Records. https://www.industrydocumentslibrary.ucsf.edu/tobacco/docs/sfln0178.

2. Marlboro Bar Nights Promotional Creative Brief. May 27 1996. Philip Morris Records. https://www.industrydocumentslibrary.ucsf.edu/tobacco/docs/rgfy0152.

3. [1997 Marlboro Racing Bar Night Program]. 1997. Philip Morris Records. https://www.industrydocumentslibrary.ucsf.edu/tobacco/docs/hkxl0172.

4. Videotaped Deposition of Steven Sampson. April 12 2002. Philip Morris Records. https://www.industrydocumentslibrary.ucsf.edu/tobacco/docs/trlp0183.

5. [Marlboro Ranch Events]. September 23 1999. Philip Morris Records. https://www.industrydocumentslibrary.ucsf.edu/tobacco/docs/qzff0155.

6. [Cold Days. Hot Nights.]. October 1999. Philip Morris Records. https://www.industrydocumentslibrary.ucsf.edu/tobacco/docs/hxmg0019.

7. McCandlish J. Service Report No. 2233 Marlboro. January 13 2000. Philip Morris Records. https://www.industrydocumentslibrary.ucsf.edu/tobacco/docs/smmv0152.

8. Permanent Younger Adult OOH Plan. 1988. RJ Reynolds Records. https://www.industrydocumentslibrary.ucsf.edu/tobacco/docs/mmdg0059.

9. Evans SY. New Brands and Strategic Research Report. Project Mp Concept Ideation Session. January 30 1985. RJ Reynolds Records. https://www.industrydocumentslibrary.ucsf.edu/tobacco/docs/jzdb0093.

10. Brainstorming NYC, 2/26. February 26 1985. RJ Reynolds Records. https://www.industrydocumentslibrary.ucsf.edu/tobacco/docs/jfcb0085.

11. Agenda. Project XG. 1985. RJ Reynolds Records. https://www.industrydocumentslibrary.ucsf.edu/tobacco/docs/hlfp0084.

12. Lindsley V. Newport's Florida Springbreak Promotion. March 07 1983. Lorillard Records. https://www.industrydocumentslibrary.ucsf.edu/tobacco/docs/gkvd0121.

13. Promotion Testing of Young Adult Concepts. July 17 1980. Joe Camel Collection. https://www.industrydocumentslibrary.ucsf.edu/tobacco/docs/gllp0094.

14. Jones ST. Idea Session. October 01 1982. Lorillard Records. https://www.industrydocumentslibrary.ucsf.edu/tobacco/docs/ggwl0126.

15. Alcome S. Exploring the World of "Virtual Reality. November 18 1994. Philip Morris Records. https://www.industrydocumentslibrary.ucsf.edu/tobacco/docs/ffym0169.

16. Marlboro Racing. 1998. Philip Morris Records. https://www.industrydocumentslibrary.ucsf.edu/tobacco/docs/gybl0006.

17. Mahan M, McComb D. Indycar II Simulator Vendor Bid Waiver. November 20 1997. Philip Morris Records. https://www.industrydocumentslibrary.ucsf.edu/tobacco/docs/tpmh0165.

18. McComb D. Interactive I/O Simulator Vendor Contract Points. November 06 1996. Philip Morris Records. https://www.industrydocumentslibrary.ucsf.edu/tobacco/docs/nlyh0175.

19. Nancrede SF. Smokers Line Up, Light Up for 500 Expo Marlboro Ride. May 27 1994. Philip Morris Records. https://www.industrydocumentslibrary.ucsf.edu/tobacco/docs/fzjl0030.

20. Marlboro Racing Bar Night Promotions. 1998. Philip Morris Records. https://www.industrydocumentslibrary.ucsf.edu/tobacco/docs/qgbh0165.

21. Huaman A. Marlboro Racing Simulators. August 21 1995. Philip Morris Records. https://www.industrydocumentslibrary.ucsf.edu/tobacco/docs/kgwv0022.

22. Marlboro Bar Night "Race for the Gear" Promotion Summary. 1998. Philip Morris Records. https://www.industrydocumentslibrary.ucsf.edu/tobacco/docs/hmdn0030.

23. Rudy M. Spin Your Wheels in Race Simulator. June 11 1993. Philip Morris Records. https://www.industrydocumentslibrary.ucsf.edu/tobacco/docs/nhjk0030.

24. [Marlboro Racing Bar Night Program Manual]. March 05 1998. Philip Morris Records. https://www.industrydocumentslibrary.ucsf.edu/tobacco/docs/lpxc0162.

25. Advanced Promotional Concepts. RJR Master Paperwork/Program Guidelines. 1989. RJ Reynolds Records. https://www.industrydocumentslibrary.ucsf.edu/tobacco/docs/gkbw0087

26. Flair Communications Agency. Competitive Update. November 15 1999. Brown & Williamson Records. https://www.industrydocumentslibrary.ucsf.edu/tobacco/docs/yndm0190.

27. Doyle P. SIM Meeting Notes from December 2, 1999. December 02 1999. Brown & Williamson Records. https://www.industrydocumentslibrary.ucsf.edu/tobacco/docs/jpyc0221.

28. Staab R. Kool Indy Simulator Recontact - Summary Report. March 29 1999. Brown & Williamson Records. https://www.industrydocumentslibrary.ucsf.edu/tobacco/docs/tmpl0225.

29. [ASU 30 Group]. 2000. Brown & Williamson Records. https://www.industrydocumentslibrary.ucsf.edu/tobacco/docs/qzbv0191.

30. 1998 Kool Racing Challenge Race Cities. January 15 1998. Brown & Williamson Records. https://www.industrydocumentslibrary.ucsf.edu/tobacco/docs/lmyl0225.

31. HORECA 1999 Kool Racing Challenge. January 01 1999. Brown & Williamson Records. https://www.industrydocumentslibrary.ucsf.edu/tobacco/docs/jjyl0225.

32. Kool SIM Tour 1999. December 15 1998. Brown & Williamson Records. https://www.industrydocumentslibrary.ucsf.edu/tobacco/docs/sxyl0225.

33. 1996 Interactive Promotion. 1996. Philip Morris Records. https://www.industrydocumentslibrary.ucsf.edu/tobacco/docs/qlld0157.

34. Media Circus. Catch That Train. November 07 1995. Philip Morris Records. https://www.industrydocumentslibrary.ucsf.edu/tobacco/docs/pmgn0004.

35. Fox M. Catch That Train Van Bar Kickoff Meeting. August 03 1995. Philip Morris Records. https://www.industrydocumentslibrary.ucsf.edu/tobacco/docs/xpdl0169.

36. 930000 MAT Van & MVP Markets. February 24 1993. Philip Morris Records. https://www.industrydocumentslibrary.ucsf.edu/tobacco/docs/rmkn0106.

37. National Van Program Overview. December 1996. Philip Morris Records. https://www.industrydocumentslibrary.ucsf.edu/tobacco/docs/xpfk0172.

38. Rubarski J. Creative Brief: 1997 Van Program. October 11 1996. Philip Morris Records. https://www.industrydocumentslibrary.ucsf.edu/tobacco/docs/hsdl0061.

39. Manager, Marlboro Vans. October 04 1996. Philip Morris Records. https://www.industrydocumentslibrary.ucsf.edu/tobacco/docs/kfpj0014.

40. Media Circus. Software Development Agreement. September 15 1995. Philip Morris Records. https://www.industrydocumentslibrary.ucsf.edu/tobacco/docs/kjhy0009.

41. Catch That Train Project. September 15 1995. Philip Morris Records. https://www.industrydocumentslibrary.ucsf.edu/tobacco/docs/lmfp0021.

42. Draft Worldwide. 2003 Bar Program Experiential Concepts. August 08 2002. RJ Reynolds Records. https://www.industrydocumentslibrary.ucsf.edu/tobacco/docs/fgyg0190.

43. September Moon Production Network. "No Bull, No Boundaries" VR Promotion. September 07 2000. RJ Reynolds Records. https://www.industrydocumentslibrary.ucsf.edu/tobacco/docs/rfgd0190.

44. Winston 2001 Bar Program. No Bull. No Boundaries. August 11 2000. RJ Reynolds Records. https://www.industrydocumentslibrary.ucsf.edu/tobacco/docs/kgjg0186.

45. Request for Proposal. Winston Bar Program. 2001. RJ Reynolds Records. https://www.industrydocumentslibrary.ucsf.edu/tobacco/docs/hpjd0190.

46. Marlboro Interactive Kiosk. June 05 2001. Philip Morris Records. https://www.industrydocumentslibrary.ucsf.edu/tobacco/docs/mfwm0218.

47. Arcade Planet. Proposed Philip Morris Touchscreen Game Console and Software. April 18 2001. Philip Morris Records. https://www.industrydocumentslibrary.ucsf.edu/tobacco/docs/zjbm0167.

48. Levan S. Marlboro Bar Program 2001. June 19 2001. Philip Morris Records. https://www.industrydocumentslibrary.ucsf.edu/tobacco/docs/hrkn0178.

49. [Interactive Kiosk Project Objectives]. November 20 2001. Philip Morris Records. https://www.industrydocumentslibrary.ucsf.edu/tobacco/docs/fxdm0218.

50. Leo Burnett Agency. Marlboro Winter Ranch Bar Night 1 Games Rules Poster - Non-Win. 2000. Philip Morris Records. https://www.industrydocumentslibrary.ucsf.edu/tobacco/docs/ljdc0055.

51. Winter Ranch Video Game - Script. March 2000. Philip Morris Records. https://www.industrydocumentslibrary.ucsf.edu/tobacco/docs/rqbv0036.

52. Colby B. Ultimate Powder Video Game - Contract Changes. July 28 2000. Philip Morris Records. https://www.industrydocumentslibrary.ucsf.edu/tobacco/docs/gtff0057.

53. Lieberman Research Worldwide. Assessing the Effectiveness and Impact of Kool's 2003 Play on the House Website. Final Report - Revised. December 01 2003. RJ Reynolds Records. https://www.industrydocumentslibrary.ucsf.edu/tobacco/docs/mmlx0225.

54. RJRT Brand Websites. Overview. December 12 2008. RJ Reynolds Records. https://www.industrydocumentslibrary.ucsf.edu/tobacco/docs/slbl0225.

55. Arkadium. Arkadium Company Overview. May 10 2005. RJ Reynolds Records. https://www.industrydocumentslibrary.ucsf.edu/tobacco/docs/jtcx0222.

56. Camel Event Marketing Training Manual. January 26 2006. RJ Reynolds Records. https://www.industrydocumentslibrary.ucsf.edu/tobacco/docs/kjhj0222.

57. Camel. Full Throttle. 2005. January 01 2005. RJ Reynolds Records. https://www.industrydocumentslibrary.ucsf.edu/tobacco/docs/lmmx0225.

58. Camel Casino. January 01 2004. RJ Reynolds Records. https://www.industrydocumentslibrary.ucsf.edu/tobacco/docs/qlbj0224.

59. This Report Provides Total Website Visits to Destination of Each Brand, Per Month, as Well as YTD. June 13 2007. RJ Reynolds Records. https://www.industrydocumentslibrary.ucsf.edu/tobacco/docs/nlvp0030.

60. Winston. the No Bull Oasis. August 21 2000. RJ Reynolds Records. https://www.industrydocumentslibrary.ucsf.edu/tobacco/docs/tghk0186.

61. Important Information About the Doral & Co. Free Carton Offer Trademark (for 70 Pack Seals). August 03 2000. RJ Reynolds Records. https://www.industrydocumentslibrary.ucsf.edu/tobacco/docs/jzwd0190.

62. Web Marketing Overview. July 2002. RJ Reynolds Records. https://www.industrydocumentslibrary.ucsf.edu/tobacco/docs/ftlx0224.

63. Mellen N. Confidential and proprietary information on Kool Mixx. May 26 2004. Depositions and Trial Testimony (DATTA). https://www.industrydocumentslibrary.ucsf.edu/tobacco/docs/yfpk0154.

64. Spitzer E. Notice of Intent to Initiate Enforcement Proceedings. May 07 2004. Depositions and Trial Testimony (DATTA). https://www.industrydocumentslibrary.ucsf.edu/tobacco/docs/rqjk0154.

65. Demand Response Systems. Nascar Racing Simulation Games for Use in Promotional Events. March 14 2000. RJ Reynolds Records. https://www.industrydocumentslibrary.ucsf.edu/tobacco/docs/lydv0189.

66. Demand Response Systems. A Tactical Promotional Plan Recommendation. July 14 1999. RJ Reynolds Records. https://www.industrydocumentslibrary.ucsf.edu/tobacco/docs/ghjw0186.

67. Loyalty Card Test. July 15 1999. RJ Reynolds Records. https://www.industrydocumentslibrary.ucsf.edu/tobacco/docs/nqlk0185.

68. A Doral & Co. Exclusive. October 07 1999. RJ Reynolds Records. https://www.industrydocumentslibrary.ucsf.edu/tobacco/docs/sgjw0186.

69. Weber Shandwick Worldwide. Buttoutnow.Com Update. October 03 2002. Lorillard Records. https://www.industrydocumentslibrary.ucsf.edu/tobacco/docs/mljw0006.

70. Teen H.I.P. - Stay on the Right Track. February 2002. Lorillard Records. https://www.industrydocumentslibrary.ucsf.edu/tobacco/docs/jymd0006.

71. Weber Shandwick Worldwide. Buttoutnow.Com Game Concept. March 22 2002. Lorillard Records. https://www.industrydocumentslibrary.ucsf.edu/tobacco/docs/grpd0006.

72. BSMG Worldwide. Recommendations for the Buttoutnow.Com Site. September 14 2000. Lorillard Records. https://www.industrydocumentslibrary.ucsf.edu/tobacco/docs/pxck0076.

73. Larsen Interactive. Buttoutnow.Com Website Report December 2000. Lorillard Records. https://www.industrydocumentslibrary.ucsf.edu/tobacco/docs/hjck0076.

74. Visitors to Buttoutnow.Com. December 2001. Lorillard Records. https://www.industrydocumentslibrary.ucsf.edu/tobacco/docs/fqcw0006.

75. Buttoutnow.Com Privacy Policy. September 17 2001. Lorillard Records. https://www.industrydocumentslibrary.ucsf.edu/tobacco/docs/jmjg0219.

76. BSMG Worldwide. Buttoutnow.Com Web Site Binder - for Review. December 08 1999. Lorillard Records. https://www.industrydocumentslibrary.ucsf.edu/tobacco/docs/ykgp0094.

77. Crisis Crew Game Buttoutnow.Com. October 1999. Lorillard Records. https://www.industrydocumentslibrary.ucsf.edu/tobacco/docs/jkgp0094.

78. Buttoutnow.Com. January 2000. Lorillard Records. https://www.industrydocumentslibrary.ucsf.edu/tobacco/docs/rjgp0094.

79. The Search Buttoutnow.Com. October 1999. Lorillard Records. https://www.industrydocumentslibrary.ucsf.edu/tobacco/docs/xkgp0094.

80. [Buttoutnow.com]. October 1999. Lorillard Records. https://www.industrydocumentslibrary.ucsf.edu/tobacco/docs/zjgp0094.

81. Lorillard Youth Smoking Prevention Program Teen H.I.P. Awards Detailed Program Overview. August 12 1999. Marketing to Women MSA Collection. https://www.industrydocumentslibrary.ucsf.edu/tobacco/docs/xhjx0045.

82. Bozell New York, BSMG. Lorillard Tobacco Company's Youth Smoking Prevention Program Brand Review. July 25 2000. Lorillard Records. https://www.industrydocumentslibrary.ucsf.edu/tobacco/docs/mgmm0166.

83. Y+R. YSP Online Advertising Scope and Budget Q4 2001. October 2001. Philip Morris Records. https://www.industrydocumentslibrary.ucsf.edu/tobacco/docs/pykn0167.

84. Giant Step. Philip Morris Youth Smoking Prevention. April 30 1999. Philip Morris Records. https://www.industrydocumentslibrary.ucsf.edu/tobacco/docs/krnw0155.

85. Brand Dialogue. Scope of Work Youth Smoking Prevention Media Campaign. July 14 1999. Philip Morris Records. https://www.industrydocumentslibrary.ucsf.edu/tobacco/docs/hpgf0155.

86. Giant Step. Giant Step & PM Youth Smoking Prevention. September 30 1998. Philip Morris Records. https://www.industrydocumentslibrary.ucsf.edu/tobacco/docs/mkvg0175.

87. Brand Dialogue. Philip Morris - Youth Smoking Prevention Speaker Concept. June 04 1999. Philip Morris Records. https://www.industrydocumentslibrary.ucsf.edu/tobacco/docs/khcp0169.

88. [YSP ideas]. February 1999. Philip Morris Records. https://www.industrydocumentslibrary.ucsf.edu/tobacco/docs/ffjh0041.

89. Giant Step. Youth Smoking Prevention Proposal. December 13 1998. Philip Morris Records. https://www.industrydocumentslibrary.ucsf.edu/tobacco/docs/hlfy0169.

90. Brand Dialogue. Philip Morris Youth Smoking Prevention. September 28 1998. Philip Morris Records. https://www.industrydocumentslibrary.ucsf.edu/tobacco/docs/mhjl0159.

91. Brand Dialogue. Philip Morris YSP Speaker Concept Production Addendum. June 04 1999. Philip Morris Records. https://www.industrydocumentslibrary.ucsf.edu/tobacco/docs/knyh0218.

92. Marlboro Racing Simulators. 1993. Philip Morris Records. https://www.industrydocuments.ucsf.edu/docs/lfcc0022.

93. Digital Tech F. Marlboro Racing Simulators Design & Budget Proposal. April 30 2001. Philip Morris Records. https://www.industrydocuments.ucsf.edu/docs/nhdm0218.

94. Virtual Alchemy Studios. Ultimate Powder Game Lazertron Meeting 990609. June 09 1999. Philip Morris Records. https://www.industrydocuments.ucsf.edu/docs/npxd0167.

95. Roque V. Re: Ultimate Powder II Enhancements. April 04 2001. Philip Morris Records. https://www.industrydocuments.ucsf.edu/docs/mqnd0058.

96. Roque V. [Action Items]. June 02 2001. Philip Morris Records. https://www.industrydocuments.ucsf.edu/docs/jsbc0055.

97. Marlboro Bars & Events Project List. April 16 2001. Philip Morris Records. https://www.industrydocuments.ucsf.edu/docs/rnln0178.

98. Young S. Re: Ultimate Powder II Enhancements. April 04 2001. Philip Morris Records. https://www.industrydocuments.ucsf.edu/docs/fqnd0058.

99. Young S. Ultimate Powder II Enhancements. March 30 2001. Philip Morris Records. https://www.industrydocuments.ucsf.edu/docs/kqnd0058.

100. Young S. Contract Request Form. October 26 2000. Philip Morris Records. https://www.industrydocuments.ucsf.edu/docs/ytjw0165.

101. Marlboro Bars & Events Project List. October 23 2000. Philip Morris Records. https://www.industrydocuments.ucsf.edu/docs/hymw0057.

102. Blohm SC, Petermeier NB. Video Game Purchase, Refurbishment and and Upgrade Agreement. September 07 2000. Philip Morris Records. https://www.industrydocuments.ucsf.edu/docs/khlw0165

103. Allen J, Blohm SC. Video Game Purchase, Refurbishment and Upgrade Agreement. May 24 2000. Philip Morris Records. https://www.industrydocuments.ucsf.edu/docs/ghlw0165.

104. Spring 2000 Ranch Materials. June 13 2000. Philip Morris Records. https://www.industrydocuments.ucsf.edu/docs/spkf0165.

105. Winter 2000 Ranch Materials. August 01 2000. Philip Morris Records. https://www.industrydocuments.ucsf.edu/docs/qphm0162.

106. [Bar Team Meeting Re-Cap]. July 31 2000. Philip Morris Records. https://www.industrydocuments.ucsf.edu/docs/jflj0218.

107. Young S. Contract Request Form. July 24 2000. Philip Morris Records. https://www.industrydocuments.ucsf.edu/docs/tqxm0177.

108. Bingenheimer A. 2000 Winter Ranch Timeline & Current Issues. July 13 2000. Philip Morris Records. https://www.industrydocuments.ucsf.edu/docs/rhnb0055.

109. Roque V. Status on WR Games. June 05 2001. Philip Morris Records. https://www.industrydocuments.ucsf.edu/docs/xfbc0055.

110. Roque V. [Project List]. May 21 2001. Philip Morris Records. https://www.industrydocuments.ucsf.edu/docs/zmxm0167.

111. Norrington P, Rogers J, Simmons C. Contract Approval Form Star Shooter 2000 Video Game. May 02 2000. Philip Morris Records. https://www.industrydocuments.ucsf.edu/docs/hhlw0165.

112. Leo Burnett Agency. Marlboro Winter Ranch Ultimate Powder Footer. 1999. Philip Morris Records. https://www.industrydocuments.ucsf.edu/docs/frwp0058.

113. Fiumara MA. Re: 2000 Materials 4-18-00.Xls. April 18 2000. Philip Morris Records. https://www.industrydocuments.ucsf.edu/docs/kzwf0057.

114. Barr R. Vsi Fax Lazertron Shipment Status. February 2000. Philip Morris Records. https://www.industrydocuments.ucsf.edu/docs/pyhj0064.

115. 99 Rock the Marlboro Ranch Materials. 1998. Philip Morris Records. https://www.industrydocuments.ucsf.edu/docs/xxhy0067.

116. Petermeier NB. Fax Game Decals. August 12 1999. Philip Morris Records. https://www.industrydocuments.ucsf.edu/docs/sgbb0039.

117. Lazertron. Difference to Artwork and Decals from Original Ranch Party. August 09 1999. Philip Morris Records. https://www.industrydocuments.ucsf.edu/docs/rgbb0039.

118. Allen J. Fax Update on Cabinets. August 09 1999. Philip Morris Records. https://www.industrydocuments.ucsf.edu/docs/kqpf0064.

119. Lazertron. [Task List]. June 09 1999. Philip Morris Records. https://www.industrydocuments.ucsf.edu/docs/khpy0164.

120. Marlboro Winter Ranch Bar Night 1&2 Ultimate Powder Game 2000. Philip Morris Records. https://www.industrydocuments.ucsf.edu/docs/kjgj0064.

121. Leo Burnett Agency. Play Marlboro Ultimate Powder. July 1999. Philip Morris Records. https://www.industrydocuments.ucsf.edu/docs/fymk0025.

122. Leo Burnett Agency. Marlboro Ultimate Powder. July 1999. Philip Morris Records. https://www.industrydocuments.ucsf.edu/docs/pgbb0039.

123. Capps Digital Mediahive Remote Approval Ultimate Powder Game (41513). June 01 1999. Philip Morris Records. https://www.industrydocuments.ucsf.edu/docs/ykxd0167.

124. Ward B. Winter Ranch Lazertron Call. May 27 1999. Philip Morris Records. https://www.industrydocuments.ucsf.edu/docs/slpy0164.

125. Bayliss E. Marlboro - Lazertron Game Re-Route. March 11 1999. Philip Morris Records. https://www.industrydocuments.ucsf.edu/docs/ztvh0025.

126. Bayliss EL. Marl. Star Shooter. March 11 1999. Philip Morris Records. https://www.industrydocuments.ucsf.edu/docs/ftyw0033.

127. Shoot a Star Game - Changes 990223. February 23 1999. Philip Morris Records. https://www.industrydocuments.ucsf.edu/docs/pxfv0020.

128. Altschul SE. Bar Materials Issues. February 19 1999. Philip Morris Records. https://www.industrydocuments.ucsf.edu/docs/ggpg0033.

129. Altschul SE. Lazertron Visit. January 08 1999. Philip Morris Records. https://www.industrydocuments.ucsf.edu/docs/xhnf0033.

130. Kelly B. Philip Morris 'Star Shooter' Game Hardware Specification. January 05 1999. Philip Morris Records. https://www.industrydocuments.ucsf.edu/docs/lfyd0025.

131. Petermeier N. Facsimile Transmittal Sheet Purchase Agreement. January 04 1999. Philip Morris Records. https://www.industrydocuments.ucsf.edu/docs/ytgf0155.

132. Lazertron. 'Star Shooter' Software Specification. January 1999. Philip Morris Records. https://www.industrydocuments.ucsf.edu/docs/gllp0027.

133. Lazertron, Philip Morris Incorporated. Purchase Agreement. January 15 1999. Philip Morris Records. https://www.industrydocuments.ucsf.edu/docs/ptgf0155.

134. 2002 Bar Program 2001. Philip Morris Records. https://www.industrydocuments.ucsf.edu/docs/spcm0218.

135. 20020000 Bar Program. 2002. Philip Morris Records. https://www.industrydocuments.ucsf.edu/docs/nrbm0218.

136. Philip Morris USA. Request for Proposal - Marlboro Bar Program Interactive Kiosk. November 20 2001. Philip Morris Records. https://www.industrydocuments.ucsf.edu/docs/trvc0055.

137. Arbetter L. Request for Proposal - Marlboro Bar Program Interactive Kiosk. November 20 2001. Philip Morris Records. https://www.industrydocuments.ucsf.edu/docs/srvc0055.

138. Sampson SF. Fw: Semi-Permanent Merchandiser Proposal. October 23 2001. Philip Morris Records. https://www.industrydocuments.ucsf.edu/docs/xkgm0057.

139. Bingenheimer A, Mark K, Roque V. Marlboro Winter Ranch 2003 Creative Look. August 30 2001. Philip Morris Records. https://www.industrydocuments.ucsf.edu/docs/pyln0178.

140. Marlboro Bars & Events Project List. June 11 2001. Philip Morris Records. https://www.industrydocuments.ucsf.edu/docs/smnn0178.

141. PM New Ground. June 2001. Philip Morris Records. https://www.industrydocuments.ucsf.edu/docs/jhfv0178.

142. Leo Burnett Agency. Marlboro Bar Program 2001. June 19 2001. Philip Morris Records. https://www.industrydocuments.ucsf.edu/docs/lffd0178.

143. Affinity Management Group. Status Report. April 20 2001. Philip Morris Records. https://www.industrydocuments.ucsf.edu/docs/myjn0178.

144. Bingenheimer A. [Action Items]. March 23 2001. Philip Morris Records. https://www.industrydocuments.ucsf.edu/docs/tsdm0218.

145. Marlboro Bar Program Interactive Kiosk RFP Comparison as of 20010105. January 05 2001. Philip Morris Records. https://www.industrydocuments.ucsf.edu/docs/thdm0218.

146. Interactive Kiosk Status. January 2002. Philip Morris Records. https://www.industrydocuments.ucsf.edu/docs/gfnn0178.

147. Catch That Train Video Game. April 1995. Philip Morris Records. https://www.industrydocuments.ucsf.edu/docs/rfjf0170.

148. Motley E. Video Games Specs. October 18 1995. Philip Morris Records. https://www.industrydocuments.ucsf.edu/docs/sxvk0157.

149. Motley E. Video Games for Project Thunder. August 28 1995. Philip Morris Records. https://www.industrydocuments.ucsf.edu/docs/slpb0167.

150. Gross D. Video Game Space. July 06 1995. Philip Morris Records. https://www.industrydocuments.ucsf.edu/docs/fgfg0076.

151. Merhige C. Video Game. July 06 1995. Philip Morris Records. https://www.industrydocuments.ucsf.edu/docs/hnpb0167.

152. Trojanowski J. Video Game Space. July 06 1995. Philip Morris Records. https://www.industrydocuments.ucsf.edu/docs/prpb0167.

153. Gross D. Video Game Machines. May 30 1995. Philip Morris Records. https://www.industrydocuments.ucsf.edu/docs/ffcc0220.

154. Digital Tech Frontier and Ftd Schedule Philip Morris Prototype 1.O Preliminary Timeline. January 07 2002. Philip Morris Records. https://www.industrydocuments.ucsf.edu/docs/zzcc0055.

155. Blair C. Racing Simulators for Racing School. January 26 2002. Philip Morris Records. https://www.industrydocuments.ucsf.edu/docs/zxvm0057.

156. Jochim S. Re: Fw: Timeline. January 22 2002. Philip Morris Records. https://www.industrydocuments.ucsf.edu/docs/gypl0057.

157. Sampson S. Simulator Development Contract. January 04 2002. Philip Morris Records. https://www.industrydocuments.ucsf.edu/docs/qzcc0055.

158. Jochim S. Re: Contract. November 05 2001. Philip Morris Records. https://www.industrydocuments.ucsf.edu/docs/zmnn0178.

159. Sampson SF. Simulators. September 27 2001. Philip Morris Records. https://www.industrydocuments.ucsf.edu/docs/xzcc0055.

160. Sampson S. Digital Tech Frontier Contract. September 21 2001. Philip Morris Records. https://www.industrydocuments.ucsf.edu/docs/mncm0167.

161. Blair C. Marlboro Racing Simulators Summary. September 13 2001. Philip Morris Records. https://www.industrydocuments.ucsf.edu/docs/jjcm0218.

162. Marlboro Bars & Events Project List. May 21 2001. Philip Morris Records. https://www.industrydocuments.ucsf.edu/docs/mmln0178.

163. Digital Tech Frontier. Philip Morris Racing Upgrade 2001. 2001. Philip Morris Records. https://www.industrydocuments.ucsf.edu/docs/qscc0055.

164. Keim T, Levan S, Sampson S, Smith D. Appropriation Request Marlboro Racing Simulators. September 04 2001. Philip Morris Records. https://www.industrydocuments.ucsf.edu/docs/ggwm0218.

165. Sampson S. Racing Simulators. September 18 2001. Philip Morris Records. https://www.industrydocuments.ucsf.edu/docs/kldm0218.

166. DTF Contract Services. September 2001. Philip Morris Records. https://www.industrydocuments.ucsf.edu/docs/xjnn0178.

167. Jochim S. [Racing simulator contract]. September 07 2000. Philip Morris Records. https://www.industrydocuments.ucsf.edu/docs/nzff0057.

168. Marlboro Bar Program Racing School Simulator Proposal. December 2001. Philip Morris Records. https://www.industrydocuments.ucsf.edu/docs/hlhm0167.

169. Marlboro Events 990000 Racing Budget. December 07 1999. Philip Morris Records. https://www.industrydocuments.ucsf.edu/docs/rzmf0218.

170. DTF Contract Recommendations. June 2001. Philip Morris Records. https://www.industrydocuments.ucsf.edu/docs/tscm0218.

171. Racing Simulators History. 2001. Philip Morris Records. https://www.industrydocuments.ucsf.edu/docs/nrcc0055.

172. Simulator Topline. 2000. Philip Morris Records. https://www.industrydocuments.ucsf.edu/docs/zscc0055.

173. 970000 Race for the Gear Events. February 19 1997. Philip Morris Records. https://www.industrydocuments.ucsf.edu/docs/tpvj0025.

174. [1998 Marlboro Racing Bar Night Program]. March 05 1998. Philip Morris Records. https://www.industrydocuments.ucsf.edu/docs/lpxc0162.

175. Lab Safety Supply. Marlboro Racing Bar Night Promotions Race for the Gear. 1998. Philip Morris Records. https://www.industrydocuments.ucsf.edu/docs/qgbh0165.

176. Degiorgio F. Agreement. January 28 1997. Philip Morris Records. https://www.industrydocuments.ucsf.edu/docs/phvf0071.

177. Interactive I. O. Virtual Vehicle Simulator. 1997. Philip Morris Records. https://www.industrydocuments.ucsf.edu/docs/hjnf0033.

178. Y+R. YSP Interactive Opportunities. May 30 2001. Philip Morris Records. https://www.industrydocuments.ucsf.edu/docs/ztbf0175.

179. Taylor N. Document regarding: Internet Advertising developed by Y&R for YSP. May 01 2002. Philip Morris Records. https://www.industrydocuments.ucsf.edu/docs/gfgp0180.

180. Contract Request Form Attachment. Philip Morris Records. https://www.industrydocuments.ucsf.edu/docs/fppw0064.

181. Giant Step. Philip Morris USA Youth Smoking Prevention Start Talking and Tabs Enhancement to Pmusa.Com Proposal. January 18 2002. Philip Morris Records. https://www.industrydocuments.ucsf.edu/docs/hpwm0167.

182. Brosterman NA. Giant Step Meeting. September 19 2001. Philip Morris Records. https://www.industrydocuments.ucsf.edu/docs/mymx0052.

183. Giant Step. Philip Morris USA Youth Smoking Prevention (Ysp) Tabs Define Proposal. September 05 2001. Philip Morris Records. https://www.industrydocuments.ucsf.edu/docs/rxlh0165.

184. Burson-Marsteller. Youth Smoking Prevention Programs Burson-Marsteller Status Report. 2001. Philip Morris Records. https://www.industrydocuments.ucsf.edu/docs/hlgf0175.

185. Daragan KM. YSP and PM Website. August 25 2000. Philip Morris Records. https://www.industrydocuments.ucsf.edu/docs/kxvw0175.

186. Caputo CW. Website Projects: Contracts to Be Drafted. March 27 2000. Philip Morris Records. https://www.industrydocuments.ucsf.edu/docs/sjvp0076.

187. Scism AK. Philip Morris Web. January 04 2000. RJ Reynolds Records. https://www.industrydocuments.ucsf.edu/docs/pzng0011.

188. Dowdell M. Giant Step Contract Request. August 10 1999. Philip Morris Records. https://www.industrydocuments.ucsf.edu/docs/hfyw0155.

189. Diamond F. Results from Giant Step's Research on PM Youth Smoking Prevention 'Cool Zone' Tweens and Influencer Web Sites. April 12 1999. Philip Morris Records. https://www.industrydocuments.ucsf.edu/docs/nnlm0159.

190. Bring MH. [Congratulations and thank you]. January 20 1997. Philip Morris Records. https://www.industrydocuments.ucsf.edu/docs/yjnn0063.

191. Giant Step. Philip Morris Youth Smoking Prevention Research Recommendation for Online Communication. March 10 1999. Philip Morris Records. https://www.industrydocuments.ucsf.edu/docs/zlhp0169.

192. Philip Morris Internet Project Status Report as of 20000614. June 14 2000. Philip Morris Records. https://www.industrydocuments.ucsf.edu/docs/myxx0172.

193. YSP Recommendations Creative Concepts. April 13 2000. Philip Morris Records. https://www.industrydocuments.ucsf.edu/docs/mzkw0178.

194. Philip Morris USA Ebusiness Strategy. April 12 2000. Philip Morris Records. https://www.industrydocuments.ucsf.edu/docs/xzvb0152.

195. Kirschbaum M. Luminant Contract Request. February 08 2000. Philip Morris Records. https://www.industrydocuments.ucsf.edu/docs/mtcy0167.

196. Woodward SM. [YSP online program status]. July 26 1999. Philip Morris Records. https://www.industrydocuments.ucsf.edu/docs/jllj0220.

197. Harel I. Thanks for Signing Up. March 31 1999. Philip Morris Records. https://www.industrydocuments.ucsf.edu/docs/prcy0023.

198. [Review of websites]. February 1999. Philip Morris Records. https://www.industrydocuments.ucsf.edu/docs/ffjh0041.

199. Sansone J. YSP Monthly Status Report Communication Group 990200. February 02 1999. Philip Morris Records. https://www.industrydocuments.ucsf.edu/docs/myxp0169.

200. Philip - Morris YSP Online Scope of Work: Phase I. January 1999. Philip Morris Records. https://www.industrydocuments.ucsf.edu/docs/ljgp0169.

201. Sansone J. YSP 99 Communications Plan. January 28 1999. Philip Morris Records. https://www.industrydocuments.ucsf.edu/docs/nfcb0162.

202. YSP Media Content Status as of 990108. January 08 1999. Philip Morris Records. https://www.industrydocuments.ucsf.edu/docs/nscp0169.

203. Y+R. Philip Morris YSp 99 Kick-Off Discussion. January 06 1999. Philip Morris Records. https://www.industrydocuments.ucsf.edu/docs/rrfp0169.

204. YSP Communications Group First Quarter 99 Objectives. April 1999. Philip Morris Records. https://www.industrydocuments.ucsf.edu/docs/znjg0167.

205. Eisen K, Sansone J. PM USA Youth Smoking Prevention. November 25 1998. Philip Morris Records. https://www.industrydocuments.ucsf.edu/docs/gljv0172.

206. Martinez V, Vanpelt M. [Thank you again]. September 29 1998. Philip Morris Records. https://www.industrydocuments.ucsf.edu/docs/nhjl0159.

207. Brand Dialogue. Scope of Work Youth Smoking Prevention (YSP) - Phase 2. June 04 1999. Philip Morris Records. https://www.industrydocuments.ucsf.edu/docs/jghp0169.

208. Introduction to IP Media. April 14 2000. Philip Morris Records. https://www.industrydocuments.ucsf.edu/docs/pgmy0152.

209. Y+R. Y&R Agenda Internetwork Session. October 19 1998. Philip Morris Records. https://www.industrydocuments.ucsf.edu/docs/kkjy0066.

210. What Are We Looking for Interactive Agencies. October 1998. Philip Morris Records. https://www.industrydocuments.ucsf.edu/docs/gzfp0169.

211. Dowdell M. Website Objectives. September 22 1998. Philip Morris Records. https://www.industrydocuments.ucsf.edu/docs/qhhp0169.

212. Sansone J. Youth Smoking Prevention WWW Project: Next Steps. August 26 1998. Philip Morris Records. https://www.industrydocuments.ucsf.edu/docs/yhdp0169.

213. Cormier BM. Home Video Games and Arcade Video Games. March 12 1990. Philip Morris Records. https://www.industrydocuments.ucsf.edu/docs/mgxk0114.

214. Barcelou DM. Contract. Driver Seat Philip Morris - 900000. November 10 1989. Philip Morris Records. https://www.industrydocuments.ucsf.edu/docs/yfkf0022.

215. Baker D. Simulator. October 09 1989. Philip Morris Records. https://www.industrydocuments.ucsf.edu/docs/krky0024.

216. Barcelou DM. [This will confirm our telephone coversation]. August 14 1989. Philip Morris Records. https://www.industrydocuments.ucsf.edu/docs/nrky0024.

217. Dangoor D. Contract Aproval Request. 1990. Philip Morris Records. https://www.industrydocuments.ucsf.edu/docs/tylv0022.

218. Barcelou DM. Philip Morris Incorporated Driver Seat Inc. Simulator Agreement. July 07 1989. Philip Morris Records. https://www.industrydocuments.ucsf.edu/docs/trky0024.

219. Baker D. [This letter represents a commitment]. June 12 1989. Philip Morris Records. https://www.industrydocuments.ucsf.edu/docs/xyjb0022.

220. Bourdon DI. [Per our phone call this morning]. June 08 1989. Philip Morris Records. https://www.industrydocuments.ucsf.edu/docs/xybl0019

221. Baker D. [The following is an outline of the agreements]. May 19 1989. Philip Morris Records. https://www.industrydocuments.ucsf.edu/docs/xrky0024

222. Proposed Terms of 1989 Driver Seat Philip Morris Agreement. 1989. Philip Morris Records. https://www.industrydocuments.ucsf.edu/docs/hrky0024.

223. Sports Consultants Intl. Sponsorship Proposal for the 1989 Driver Seat Debut and the 1990 Drivers Seat Season. January 25 1989. Philip Morris Records. https://www.industrydocuments.ucsf.edu/docs/rgvb0017.

224. Rek Room; 2000 Objectives. 2000. Brown & Williamson Records. https://www.industrydocuments.ucsf.edu/docs/thcb0172.

225. Analytic Insight Inc. Rek Mystery Audit Report; Kool Rek; Max 2001. September 30 2000. Brown & Williamson Records. https://www.industrydocuments.ucsf.edu/docs/qhfg0191.

226. Tortora A. Cigarette Marketing Targets Night Life. September 26 1999. RJ Reynolds Records. https://www.industrydocuments.ucsf.edu/docs/gqck0091.

227. Horeca Kool Music - Mixx. August 10 1999. Brown & Williamson Records. https://www.industrydocuments.ucsf.edu/docs/xxgm0190.

228. Colline L. Price Quotation for Kool Racing Challenge Handout. May 13 1999. Brown & Williamson Records. https://www.industrydocuments.ucsf.edu/docs/ksyx0081.

229. Agreement for Services. April 1999. Brown & Williamson Records. https://www.industrydocuments.ucsf.edu/docs/ytpb0191.

230. Annex F, Sim Tour Budget Based on 4 Nights a Week. Brown & Williamson Records. https://www.industrydocuments.ucsf.edu/docs/szyx0081.

231. Kool Racing Challenge Operational Review - 1998. 1998. Brown & Williamson Records. https://www.industrydocuments.ucsf.edu/docs/zmxm0190.

232. Fabulous 5. Five New Ideas That We Have 90% Confidence Will Significantly Impact the Business. February 22 2002. RJ Reynolds Records. https://www.industrydocuments.ucsf.edu/docs/qfxx0224.

233. No Bull, No Boundaries. Bar Program. August 31 2000. RJ Reynolds Records. https://www.industrydocuments.ucsf.edu/docs/jqxg0186.

234. Gyro Worldwide Adv. Bar Program Ideas. October 02 2000. RJ Reynolds Records. https://www.industrydocuments.ucsf.edu/docs/lkdd0190.

235. Davies M. Winston Skydiving VR Experience. September 21 2000. RJ Reynolds Records. https://www.industrydocuments.ucsf.edu/docs/rndb0190.

236. Software Supply Agreement between Maelstrom Virtual Productions Limited & Customer R.J. Reynolds Tobacco Company. October 02 2000. RJ Reynolds Records. https://www.industrydocuments.ucsf.edu/docs/nfvv0190.

237. Winston Virtual Reality Script. September 19 2000. RJ Reynolds Records. https://www.industrydocuments.ucsf.edu/docs/pjgk0186.

238. Cooperman RL. Winston "No Bull, No Boundaries" Promotion. August 31 2000. RJ Reynolds Records. https://www.industrydocuments.ucsf.edu/docs/frdb0190.

239. September Moon Production Network. Group 3 Promotions. "No Bull, No Boundaries" VR Promotion. September 07 2000. RJ Reynolds Records. https://www.industrydocuments.ucsf.edu/docs/rfgd0190.

240. Roberson MT. Winston RFP's for 1400 VR Units. July 12 1995. Philip Morris Records. https://www.industrydocuments.ucsf.edu/docs/ttfk0014.

241. R.J. Reynolds Tobacco USA. Monthly Operations Letter - September 1988 September 1988. RJ Reynolds Records. https://www.industrydocuments.ucsf.edu/docs/hsfw0023.

242. Bar Program 95-96 DMA Technology Applications. June 1995. Philip Morris Records. https://www.industrydocuments.ucsf.edu/docs/ksnm0169.

243. Marlboro Bar Program 95-96. July 19 1995. Philip Morris Records. https://www.industrydocuments.ucsf.edu/docs/jhng0012.

244. Leo Burnett Agency. Service Report No. 2094 Client Philip Morris. May 15 1995. Philip Morris Records. https://www.industrydocuments.ucsf.edu/docs/hnkm0169.

245. Leo Burnett Agency. Mu Iii Catalogue CD. January 1998. Philip Morris Records. https://www.industrydocuments.ucsf.edu/docs/khhd0028.

246. Project Winston. Winston Alternative Campaigns 2/22/96. February 22 1996. RJ Reynolds Records. https://www.industrydocuments.ucsf.edu/docs/lrgg0186.

247. Carroll RC, Powell BJ. Contract No. 5114-00-00. May 20 1995. RJ Reynolds Records. https://www.industrydocuments.ucsf.edu/docs/mldh0086.

248. Davis R. The Winston/Charlotte Motor Speedway Brochure. April 03 1989. RJ Reynolds Records. https://www.industrydocuments.ucsf.edu/docs/ffkm0084.

249. Garner MA, Smith SR. Presentation Materials from Arkadium. May 11 2005. RJ Reynolds Records. https://www.industrydocuments.ucsf.edu/docs/qxkw0217.

250. Marketing It Business Solutions Major 2005 Accomplishments. November 22 2005. RJ Reynolds Records. https://www.industrydocuments.ucsf.edu/docs/fxyx0222.

251. 92 Trade Exhibit Review. April 1992. Philip Morris Records. https://www.industrydocuments.ucsf.edu/docs/zyhy0119.

252. Foster DB. Use of Sega out Run Game by Philip Morris. May 21 1990. Philip Morris Records. https://www.industrydocuments.ucsf.edu/docs/hjjj0113.

253. [Philip Morris USA exhibit]. May 1988. Philip Morris Records. https://www.industrydocuments.ucsf.edu/docs/rtkg0127.

254. Annotated. January 05 1976. Lorillard Records. https://www.industrydocuments.ucsf.edu/docs/shlg0126.

255. A Dynamic Profit Generating Promotional Opportunity. 1999. RJ Reynolds Records. https://www.industrydocuments.ucsf.edu/docs/plwf0224.

256. Cecchini L, Ott K. Papyrus. February 08 2000. RJ Reynolds Records. https://www.industrydocuments.ucsf.edu/docs/mrxh0190.

257. Forsythe A. Kool Target Users. June 13 1985. Brown & Williamson Records. https://www.industrydocuments.ucsf.edu/docs/zhwf0142.

258. [Creative positioning]. 1982. Brown & Williamson Records. https://www.industrydocuments.ucsf.edu/docs/hncb0136

259. A Marketing Publicity Program in Support of the Apollo Chilimpics. October 1982. Brown & Williamson Records. https://www.industrydocuments.ucsf.edu/docs/tzyg0142.

260. Consultative Resources. R. J. Reynolds Session June 29, 1983 (730629). the Cloister, Sea Island, Georgia. June 29 1983. RJ Reynolds Records; Minnesota Documents. https://www.industrydocuments.ucsf.edu/docs/mxfk0096.

261. [Newsletter]. January 01 1983. Lorillard Records. https://www.industrydocuments.ucsf.edu/docs/rmvh0126

262. Hammer RD. Newport - Promotional Planning Calendar. January 28 1983. Lorillard Records. https://www.industrydocuments.ucsf.edu/docs/lpcc0011.

263. Schmitz EM. [Thank you for your recent promotion suggestion]. December 09 1982. Lorillard Records. https://www.industrydocuments.ucsf.edu/docs/ptfw0113.

264. Hammer RD. [I have just received your comments]. March 16 1982. Lorillard Records. https://www.industrydocuments.ucsf.edu/docs/slpk0060.

265. White HR. Section Operations Report. March 1982. Philip Morris Records. https://www.industrydocuments.ucsf.edu/docs/qlck0130.

266. Kohl RF. Section Operations Report. March 31 1982. Philip Morris Records. https://www.industrydocuments.ucsf.edu/docs/lmhg0127.

267. Winter Ranch Games Structure & Quantities. May 1999. Philip Morris Records. https://www.industrydocuments.ucsf.edu/docs/jgwj0220.

268. 2001 Philip Morris Audit Guidelines. 2001. Philip Morris Records. https://www.industrydocuments.ucsf.edu/docs/thxd0055.

269. Marlboro Bar Night Program. 1999. Philip Morris Records. https://www.industrydocuments.ucsf.edu/docs/jhpj0014.

270. [The Marlboro Ranch]. September 23 1999. Philip Morris Records. https://www.industrydocuments.ucsf.edu/docs/qzff0155

271. Czarnecki JR. Rough Budget Numbers - Interactive Kiosk. June 08 2000. Philip Morris Records. https://www.industrydocuments.ucsf.edu/docs/zfxn0059.

272. Arcade Planet. Marlboro Kiosk. May 2000. Philip Morris Records. https://www.industrydocuments.ucsf.edu/docs/grvx0064.

273. Affinity Management Group. Marlboro Bar Program Video Game Synopsis Spring Ranch Promotional Window. 2000. Philip Morris Records. https://www.industrydocuments.ucsf.edu/docs/yqvx0064.

274. Bible MM. Marlboro Rewards. June 02 2000. Philip Morris Records. https://www.industrydocuments.ucsf.edu/docs/lldg0218.

275. Lund N, Murphy M. Marlboro 2000 Plans. October 20 1999. Philip Morris Records. https://www.industrydocuments.ucsf.edu/docs/sgjg0155.

276. Marlboro Rewards. September 13 1999. Philip Morris Records. https://www.industrydocuments.ucsf.edu/docs/gshg0155.

277. Marlboro 2000 Plans. March 20. Philip Morris Records. https://www.industrydocuments.ucsf.edu/docs/qjby0189.

278. Amell Group Brand Consulting. Marlboro Location Based Entertainment Project. April 29 1999. Philip Morris Records. https://www.industrydocuments.ucsf.edu/docs/szxg0041.

279. Mahan M, Rose C. [Agreement]. February 25 1999. Philip Morris Records. https://www.industrydocuments.ucsf.edu/docs/jjvn0073.

280. Tenbarge D. Marlboro Kiosk Contract. March 03 1999. Philip Morris Records. https://www.industrydocuments.ucsf.edu/docs/tzgp0169.

281. Tenbarge D. Contract Approval Request Marlboro Interactive Kiosk. October 14 1998. Philip Morris Records. https://www.industrydocuments.ucsf.edu/docs/pfnd0160.

282. Request for Proposal. June 01 1998. Philip Morris Records. https://www.industrydocuments.ucsf.edu/docs/ngcp0169.

283. Lalonde S. Interactive Kiosk Content. November 17 1996. Philip Morris Records. https://www.industrydocuments.ucsf.edu/docs/lpjj0046.

284. Kern D. Marlboro Bar Night Interactive Catch the Train. June 14 1995. Philip Morris Records. https://www.industrydocuments.ucsf.edu/docs/jthp0217.

285. Cyberevent Group. Philip Morris USA Van Program Interactive Project Proposal for Trailer Based Interactive Adventure Experience. June 05 1995. Philip Morris Records. https://www.industrydocuments.ucsf.edu/docs/kxcb0012.

286. McDonough T. DMA Considerations for Thunder Promotions. June 01 1995. Philip Morris Records. https://www.industrydocuments.ucsf.edu/docs/jjym0169.

287. New Media Review. May 19 1995. Philip Morris Records. https://www.industrydocuments.ucsf.edu/docs/kspc0157.

288. Don Jagoda Associates. A Continuity Promotion Marketing Plan for Marlboro. March 02 1995. Philip Morris Records. https://www.industrydocuments.ucsf.edu/docs/yldg0162.

289. Media Department Technology Review. April 1994. Philip Morris Records. https://www.industrydocuments.ucsf.edu/docs/gygc0157.

290. Capreol GR. New Media Interactive Kiosk. March 21 1994. Philip Morris Records. https://www.industrydocuments.ucsf.edu/docs/qskl0033.

291. Kuffler M. DMA Concerns. May 05 1995. Philip Morris Records. https://www.industrydocuments.ucsf.edu/docs/zrnm0169.

292. The Promise of Digital Interactive Multimedia New Options for Philip Morris. March 11 1994. Philip Morris Records. https://www.industrydocuments.ucsf.edu/docs/fkcx0122.

293. Leo Burnett Agency. The Promise of Digital Interactive Multimedia New Options for Philip Morris. March 11 1994. Philip Morris Records. https://www.industrydocuments.ucsf.edu/docs/qhmj0121.
